# RNA Signaling in Pulmonary Arterial Hypertension—A Double-Stranded Sword

**DOI:** 10.3390/ijms21093124

**Published:** 2020-04-28

**Authors:** Helena A. Turton, A. A. Roger Thompson, Laszlo Farkas

**Affiliations:** 1 Infection, Immunity and Cardiovascular Disease, University of Sheffield, Sheffield S10 2RX, UK; 2 Pulmonary Vascular Diseases Unit, Royal Hallamshire Hospital, Sheffield S10 2RX, UK; 3 Department of Internal Medicine, Division of Pulmonary, Critical Care and Sleep Medicine, Davis Heart & Lung Research Institute, The Ohio State University, Columbus, OH 43210, USA

**Keywords:** pulmonary arterial hypertension, Toll-like receptor, TLR3, double-stranded RNA, RNA sensors, vascular remodeling

## Abstract

Recognition of and response to pathogens and tissue injury is driven by the innate immune system via activation of pattern recognition receptors. One of the many patterns recognized is RNA and, while several receptors bind RNA, Toll-like receptor 3 (TLR3) is well placed for initial recognition of RNA molecules due to its localization within the endosome. There is a growing body of work describing a role for TLR3 in maintenance of vascular homeostasis. For example, TLR3 deficiency has been shown to play repair and remodeling roles in the systemic vasculature and in lung parenchyma. A hallmark of pulmonary arterial hypertension (PAH) is pulmonary vascular remodeling, yet drivers and triggers of this remodeling remain incompletely understood. Based on its role in the systemic vasculature, our group discovered reduced endothelial TLR3 expression in PAH and revealed a protective role for a TLR3 agonist in rodent models of pulmonary hypertension. This review will provide an overview of RNA signaling in the vasculature and how it relates to PAH pathobiology, including whether targeting double-stranded RNA signaling is a potential treatment option for PAH.

## 1. Introduction

Tremendous effort has been put forward into understanding dysregulated pathways in pulmonary arterial hypertension (PAH) and these investigations span numerous topics including genetics, growth factors, metabolism and inflammation [1,2,3]. Links between inflammation and PAH are strong, although the role of the innate immune system in the initiation or propagation of vascular remodeling remains incompletely defined.

Over the past two decades, we have come to understand that innate immunity is of fundamental importance as not only the first line of defense from external pathogens, but in protection from cellular injury and maintenance of tissue homeostasis [4,5]. Stemming from Janeway’s concept of “pattern recognition receptors” (PRRs) that bind “pathogen-associated molecular patterns” (PAMPs), ongoing research has found that PRRs also recognize internal “damage-associated molecular patterns” (DAMPs) [6,7,8]. Whereas PAMPs were bits and pieces that came from processing of pathogens by the immune system, DAMPs are frequently part of the structural and functional components of our own cells and tissues, which are released during tissue injury. Extracellular DAMPs include hyaluronan, heparan sulfate, fibronectin and Tenascin C [9]. In addition to these extracellular molecules, DAMPs can also be derived from intracellular material released when cells are so severely injured that they undergo apoptosis or necrosis. DNA, including mitochondrial DNA, serves as intracellular DAMP via several PRRs [10]. Likewise, RNA has emerged as an important intracellular DAMP [11].

In recent years, Toll-like receptor 3 (TLR3) has been established as a central PRR for all sorts of RNA molecules [7,11,12,13,14,15,16,17]. Principally recognized as a viral double-stranded RNA (dsRNA) sensor, TLR3 also responds to synthetic dsRNA such as polyinosinic:polycytidylic acid [poly(I:C)] and to endogenous mRNA [11] and has been implicated in tissue repair and remodeling in a number of experimental settings [18,19,20,21]. Here, we summarize the main RNA signaling pathways and we discuss evidence for the role of these pathways in tissue repair, focusing on TLR3. We also discuss whether activation of RNA signaling could be used as a strategy to ameliorate the vascular remodeling that characterizes pulmonary arterial hypertension.

## 2. RNA Sensors

### 2.1. Toll-like Receptor 3 and Endosomal RNA Recognition

TLR3 was initially shown to sense synthetic and viral dsRNA [13]. Structural analysis of dsRNA bound to TLR3 revealed that 40–50 base-pair duplexes bind to a TLR3 dimer [22]. An alternative model suggests TLR3 dimers could also bind shorter duplexes [23], although application of various lengths of dsRNA to murine dendritic cells required duplexes of more than 90 base-pairs to invoke signaling [24]. TLR3 can also sense incomplete stem structures, such as in vitro transcribed RNAs, and is therefore capable of responding to single stranded RNA depending on its length and secondary structure [25].

An acidic environment is further a prerequisite for optimal recognition of RNA by TLR3 [26]. It is therefore not surprising that TLR3 is not primarily expressed on the cell surface but is localized within endosomes [27,28]. It is less clear whether endosomal cleavage of TLR3 is also required for downstream signaling. Following transport of full-length TLR3 from the endoplasmic reticulum, endosomal cathepsins cleave TLR3 in the absence of RNA stimulation [29,30]. Although cathepsin inhibitors reduced cleavage and TLR3 signaling in a retinal epithelial cell line [29], cathepsin inhibition had no effect on responses to poly(I:C) in immortalized lung epithelial cells [30]. In other work, co-expression of both fragments was found to be necessary for activation of reporter activity [31]. Interestingly, cathepsin inhibition altered responses of murine macrophages to other TLR3 ligands such as polyadenylic:polyuridylic acid [poly(A:U)] and double-stranded Reovirus RNA [30]. This implies that TLR3 cleavage may be important in mediating the magnitude of response to different RNAs and raises the possibility of cell- and tissue-specific consequences of TLR3 cleavage.

As hydrophilic nucleic acids cannot permeate the cell membrane, there must be ways for these molecular patterns to enter the cell. One common route to achieve transport of hydrophilic molecules into the cell is to generate membrane invaginations encapsulating the molecules to be shuttled into the cell, a process referred to as endocytosis. There are different endocytosis systems based on the underlying molecular mechanism of how endocytosis occurs. The main distinction is between the clathrin- and caveolin-mediated endocytosis, yet there are also clathrin- and caveolin-independent forms of endocytosis [32]. Delivery of nucleic acid to endosomal TLR3 has been associated with the clathrin-dependent endosome system in a process dependent upon the lipid raft protein, raftlin [28,33].

### 2.2. Other Endosomal RNA Receptors

In addition to TLR3, other TLRs (TLR7, TLR8 and, in mice, TLR13) contribute to recognition of RNA in the endosome and represent additional first responders to RNA that enters the cell by endocytosis. TLR3, TLR7 and TLR8 are trafficked to the early endosome via the chaperone Unc-93 homologue B1 (UNC93B1) [34], but the intracellular localization of TLR3 is directed by a 23 amino acid sequence in the linker region between the transmembrane domain and the Toll-Interleukin-1 receptor resistance (TIR) domain, while the domain responsible for directing TLR7 trafficking is its transmembrane domain [35]. TLR7 and TLR8 are highly homologous and form dimers in the absence of stimulation but there are differences in the type of cells in which they are expressed. Both are expressed in monocyte/macrophages, while TLR7 is found in plasmacytoid dendritic cells and TLR8 in myeloid dendritic cells [36,37]. Both respond to single stranded RNAs and activation depends upon the frequency of specific uridine-containing motifs in the RNA [38,39].

### 2.3. Cytosolic RNA Recognition

The endosome is not the only cellular compartment in which RNA is sensed; the cytosol of mammalian cells contains various receptors for RNA molecules. These receptors likely mediate synergistic and/or sequential responses to RNA together with the endosomal recognition receptors. The dominant cytosolic receptors belong to the family of RIG-I-like receptors (RLRs) that has three members: retinoid acid inducible gene-I (RIG-I), melanoma differentiation antigen 5 (MDA-5) and ‘laboratory of genetics and physiology 2’ (LGP2) [40,41,42,43]. Whereas RIG-I and MDA-5 contain caspase activating and recruitment domains (CARDs) and regulate interferon production and apoptosis, LGP2 is thought to play a regulatory role [44,45,46,47,48,49]. In fact, LGP2 sensitizes to MDA-5-induced signaling, while restricting RIG-I-induced signaling [43,50]. The importance of the cytosolic RNA receptors RIG-I and MDA-5 in immune dysregulation has been demonstrated by association of gain-of-function mutations in these receptors with the breach of distinction between self- and non-self RNAs in autoimmune conditions, including type I diabetes [51].

Another cytosolic receptor, protein kinase R (PKR), is an integrated stress response kinase which can independently induce interferon signaling upon dsRNA recognition or act synergistically with RIG-I and MDA-5 [52,53]. Beyond its canonical role as an activator of eukaryotic initiation factor 2-alpha (eIF2α), PKR regulates numerous signaling pathways (reviewed in [54]). Double-stranded RNA of over 33 base pairs is thought to be necessary to activate PKR [55] although shorter duplexes with single-stranded tails also induced PKR auto-phosphorylation [56].

A variety of other RNA-binding proteins and DExD/H box helicases have also been implicated in recognition of cytosolic RNA, for example, Leucine-Rich Repeat Flightless-Interacting Protein 1 (LRRFIP1) [57] and the 2’,5’-oligoadenylate synthetase (OAS)-RNase L pathway. These are reviewed elsewhere [58]. As we will focus on the key cytosolic and endosomal immune sensors, RNA-binding proteins with roles in the maturation of micro RNAs and in the regulation of RNA stability or structure, for example Dicer and ADAR (double-stranded RNA-specific adenosine deaminase), will also be neglected in this review. These have also been reviewed elsewhere [59,60].

## 3. RNA Signaling in the Cell

**Figure 1:**
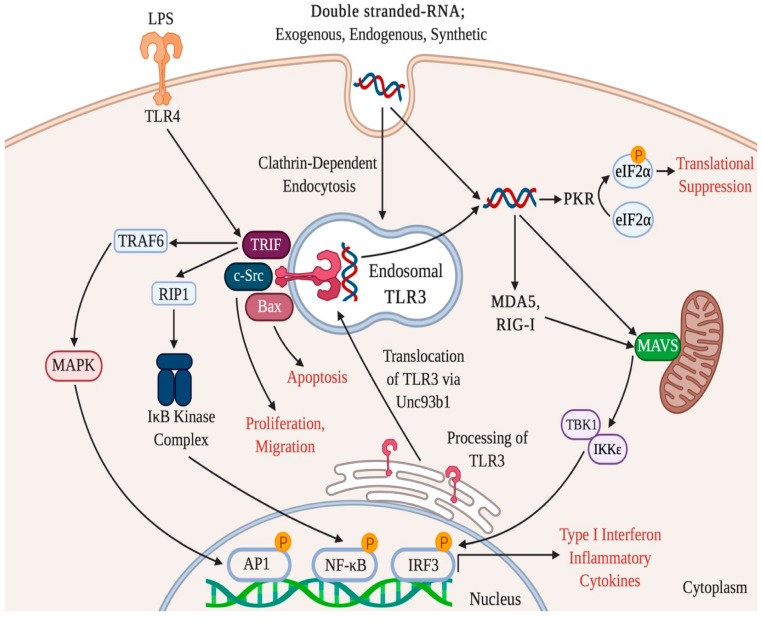
Intracellular TLR3 signaling pathways. Double stranded-RNA (dsRNA) derived from virus, mRNA liberated from necrotic cells and synthetic polyinosinic:polycytidylic acid are recognized by endosomal toll-like receptor, TLR3. TLR3 is located in acidic endosomes formed during clathrin-dependent endocytosis. TLR3 signaling occurs via TRIF, an adaptor that can also be recruited by TLR4. TRIF activates NF-κB and AP1 to induce transcription of type I interferon and pro-inflammatory cytokines. Apoptosis and proliferation can be induced by TRIF-independent TLR3 signaling via BAX and c-SRC respectively. Cytosolic receptors can respond to dsRNA independently of TLR3 or in a synergistic manner and these include retinoic acid inducible gene-I (RIG-I) and melanoma differentiation antigen 5 (MDA-5). These signal via the mitochondrial anti-viral signaling protein (MAVS) to activate interferon regulatory factor 3 (IRF3). RNA can also suppress translation via activation of protein kinase R (PKR), which in turn phosphorylates eukaryotic initiation factor 2-alpha (EIF2α). Abbreviations: TRIF, TIR-domain-containing adapter-inducing interferon-β (TRIF); TRAF6, Tumor necrosis factor receptor (TNFR)-associated factor 6; MAPK, mitogen-activated protein kinase; RIP-1, receptor-interacting protein 1; AP-1, Activator protein 1; BAX, BCL2 Associated X Protein; TBK1, TANK-binding kinase; IKK, inhibitor of nuclear factor-κB (IκB) kinase. Created with BioRender (Toronto, ON, Canada).

After initial recognition, complex and inter-linked pathways mediate the intracellular effects of RNAs. TLR agonists trigger the dimerization of Toll/interleukin 1 receptor/resistance (TIR) domains that recruit cytosolic TIR domain-containing adaptors for intracellular signaling [61]. It was originally proposed that all TLRs utilized the essential adaptor molecule myeloid differentiation primary response 88 (MyD88) [13]. However, TLR3 immune responses are mediated by TIR-domain containing adaptor protein inducing interferon beta (TRIF) [62]. TRIF links TLR3 to several downstream pathways which ultimately activate interferon regulatory factor 3 (IRF3), activator protein 1 (AP1) or NF-κB (nuclear factor kappa-light-chain-enhancer of activated B cells) transcription factors. The C-terminus of TRIF contains an interaction motif which activates members of the receptor interacting protein (RIP) family and the N-terminal region of TRIF contains three TRAF-6 binding domains [61]. Interestingly, TLR4 is the only other TLR that shares the TRIF pathway with TLR3, although TLR4 also signals via MyD88 [63]. RIP1 and TRAF6 are indispensable in TLR3-mediated NF-κB activation by inducing the liberation and translocation of NF-κB into the nucleus via the activation of the IκB kinase complex [62,64]. Nuclear accumulation of IRF3, AP1 and NF-κB results in transcription of various genes that regulate host defense and inflammation, including type I interferons (IFNs) IFN-α and IFN-β (see Figure 1).

A TRIF-independent branch of TLR3 signaling has also been described [65]. Specifically, dsRNA-stimulated dimerization of TLR3 led to transient recruitment of the proto-oncoprotein c-Src, leading to phosphorylation and subsequent redistribution of Src to lipid rafts [66]. This work linked TLR3 activation to TRIF-independent effects on cell migration and proliferation and supports a role for TLR3 in tissue repair and remodeling that is discrete from its canonical function as a viral sensor. Evidence that TLR3, and other dsRNA sensors, can respond to endogenous ligands strengthens this hypothesis and is discussed below.

## 4. TLR3 and Damage Sensing

### 4.1. Evidence for Endogenous RNA Sensing

Extending Janeway’s concept of pathogen recognition, Matzinger proposed that the innate immune system has a broader role in sensing danger, rather than only pathogenic material [67]. Danger signals include endogenous molecules released by damaged cells or tissue which are recognized by PRRs. Evidence that activation of TLR9 by endogenous DNA containing CpG motifs supported a role for TLRs in endogenous danger signals [68]. Subsequently, TLR3 was shown to be activated by in vitro transcribed mRNA and RNA associated with necrotic cells [11]. Other work demonstrated that cytokine release from peritoneal macrophages stimulated by necrotic neutrophils was dependent upon TLR3 and could be attenuated by the addition of RNAse [15]. Although initial work on DAMPs focused on maladaptive responses that induce auto-immunity or tissue graft rejection, these danger or damage sensors are now also known to be important in the activation of tissue repair pathways and the maintenance of tissue homeostasis.

### 4.2. TLR3 and Tissue Repair

Evidence for TLR3-mediated tissue repair or protection following injury spans several organs and models. Healing of full-thickness sterile wounds was delayed in TLR3 knockout mice [69] and wound-induced hair neogenesis (WIHN), the regeneration of hair follicles and sebaceous glands after wounding, was also impaired [20]. In the latter study, addition of a single local dose of dsRNA, 3 days after skin wounding in wild-type mice, increased the number of regenerated follicles, while addition of RNAse III to the site reduced the number of follicles [20]. TLR3 activation by dsRNA induced aldehyde dehydrogenase 1 family member A3 (ALDH1A3), an enzyme which converts retinol to retinoic acid in keratinocytes, and accumulation of RA was required for WIHN [70].

TLR3 also controls tissue remodeling in the lungs. In a bleomycin-induced model of pulmonary fibrosis, TLR3 knockout mice experienced worse outcomes, with increased pro-fibrotic cytokine release, collagen deposition and higher mortality compared to wild-type mice [19]. Interestingly, idiopathic pulmonary fibrosis patients with the Leu412Phe polymorphism in TLR3 (associated with attenuated IRF3 signaling [71]) had accelerated lung function decline, suggesting cross-species conservation of TLR3’s role in regulating fibrosis [19].

Based on the close connection between lung parenchymal fibrosis and lung vascular injury and reorganization [72,73], it is not surprising that TLR3 has also been implicated in vascular homeostasis. Cole et al. demonstrated that TLR3 deficiency in mice was associated with enhanced elastic lamina damage following carotid artery injury [18]. Poly(I:C) significantly reduced neointima formation in response to the carotid collar injury and these vasoprotective effects of poly(I:C) were ablated in TLR3-deficent mice [18]. Furthermore, hypercholesterolaemic (Apolipoprotein E^-^/^-^) mice crossed with TLR3 knockout mice developed early onset atherosclerosis [18]. Although the exact mechanism underpinning these beneficial effects is still unclear, the induction of anti-inflammatory cytokines is one possibility. In a further model of systemic vessel injury, the hind limb ischemia model, angiogenesis induced by shockwave therapy was abolished in TLR3-deficient mice [74]. Interestingly, supernatant of endothelial cells treated with shockwave therapy had higher levels of RNA than untreated cells, providing evidence that this technique promoted RNA release and that this was required for TLR3 activation [74]. However, the link between TLR3 and regulation of angiogenesis is controversial. Short RNA sequences, such as non-targeted small interfering RNA (siRNA), were found to inhibit angiogenesis in a TLR3-dependent manner in models of choroidal neovascularization [17] and in the hind limb ischemia model [16]. These sequences were as effective as siRNA targeting the pro-angiogenic growth factor, vascular endothelial growth factor (VEGF-A) and did not require internalization of the RNA [16]. On the other hand, TLR3 engagement promoted expression of VEGF-A in fibroblasts [75] and prostate cancer cell lines [76]. These findings suggest a homeostatic role for TLR3 activation in angiogenesis, with a balance between suppression of the process by exogenous RNA administration and DAMP-mediated activation of TLR3 contributing to initiation of angiogenesis.

In the heart, the regeneration of neonatal mouse heart tissue was impaired in TLR3 deficient animals following myocardial infarction [77]. This phenotype was attributed to reduced proliferation of TLR3 deficient cardiomyocytes while poly(I:C) promoted glycolysis and proliferation in a YAP1-dependent manner [77]. Other cardiac roles for TLR3 have been reported in post-viral infection myocardial injury [78,79] and post-ischemia/reperfusion (I/R) injury [80]. Hardarson et al. (2007) reported that TLR3 deficient mice were more susceptible to encephalomyocarditis virus (EMCV), with increased mortality compared to wild-type [78]. In EMCV-infected mice, serum analysis showed higher cardiac troponin I levels in TLR3^-/-^ mice, compared to TLR3^+/+^ mice, suggesting a direct EMCV-induced myocardial injury. In a different model that used heart-passaged coxsackievirus B3 (CVB3), TLR3 deficient mice were less effective at eliminating the virus in the acute phase and developed more severe chronic myocarditis with greater cardiac dysfunction, inflammation and fibrosis than wild-type mice [79]. In comparison to these protective effects of myocardial TLR3 against virus, Chen et al. (2014) demonstrated that both TLR3 and TRIF deficient mice exhibited significant reduced infarct size and improved left ventricular function, 24 h after I/R injury [80]. TRIF deficient mice had marked decreases in myocardial caspase 3 activity, indicative of apoptosis, after I/R injury. Interestingly, while TLR3 deficient mouse cardiomyocytes had reduced necrotic cell-induced cytokine production compared to wild-type cells, there was no in vivo evidence of altered inflammatory cell recruitment or cytokines following I/R injury in TLR3 or TRIF deficient mice [80].

A further interesting observation in cardiomyocytes demonstrated that TLR3 regulation of tissue homeostasis may also occur without dsRNA stimulation. Gao et al. (2019) revealed the role for TLR3 in the regulation of potassium channels and cardiac electrophysiological homeostasis that was not dependent on downstream immune signals. TLR3-deficient mice had prolonged QT intervals, associated with reduced amplitude of current intensity compared to control cells and this was independent of MyD88 and TRIF signaling and unaffected by poly(I:C) stimulation [81]. TLR3 predominantly resided in the endoplasmic reticulum of cardiomyocytes, which express little of the UNC93B1 chaperone protein, and was found to be important for potassium channel synthesis [81]. This work indicates a tissue-specific role in the context of limited endosomal TLR3 trafficking, but whether this is important in other cells or whether TLR3 is involved in the stabilization of other proteins remains unknown.

The work above implicates TLR3 as a regulator of tissue repair in several organs and in the systemic vasculature. Our work extended these observations to the context of pulmonary vascular remodeling in pulmonary hypertension.

## 5. TLR3 Signaling in PAH

Pulmonary hypertension (PH) is classified into 5 groups according to clinical and pathological features [82]. PAH (or Group 1) is rare disorder that is specifically characterized by sustained elevation of mean pulmonary artery pressure and pulmonary vascular resistance that ultimately precipitates right heart failure. PAH can be associated with connective tissue disease or congenital heart defects or it may be idiopathic. Irrespective of the disease driver, patients share pathophysiological processes such as sustained vasoconstriction and excessive pulmonary vascular remodeling. PAH can be referred to as a pan vasculopathy as all layers (endothelial, fibroblast and smooth muscle cells) of the vessel wall exhibit pathology including cancer-like phenotypes of excessive proliferation and apoptosis resistance [83].

TLRs have been implicated in PAH. TLR4 is the most abundantly expressed TLR receptor in human pulmonary artery smooth muscle cells (PASMCs) [84] and studies have reported that TLR4 deficient mice are protected against chronic hypoxia-induced PH [85]. Activation of TLR4 signaling using high mobility group box 1 (HMGB1), exacerbated PH in this model [86] and has been linked to suppression of bone-morphogenetic protein receptor 2 (BMPR2) signaling [87]. Variants in the *BMPR2* gene are the commonest genetic cause of PAH [88] and reduced signaling is also reported in lung tissue from patients without *BMPR2* mutations [89]. Reduced BMPR2 signaling promotes cytokine release from PASMCs following stimulation with lipopolysaccharide, the canonical TLR4 ligand [90] and has also been linked to abnormal TLR9 responses to mitochondrial DNA in pulmonary artery endothelial cells (PAECs) [91]. These findings affirm the importance of interaction between endogenous ligands and TLRs in pulmonary hypertension pathophysiology but in this review, we will focus on RNA signaling via TLR3.

We revealed evidence of loss of TLR3 expression in pulmonary artery endothelial cells (PAECs) from patients with PAH [21]. Whole lung TLR3 expression was reduced by day 21 in the chronic hypoxia and SU5416 rat model of PH and the proportion of TLR3 positive intimal cells was reduced. Using the same disease inducers (hypoxia and SU5416), TLR3 knockout mice developed more severe disease, with higher right ventricular systolic pressure (RVSP) and evidence of greater small pulmonary artery muscularization [21]. CRISPR/cas9-mediated reduction in TLR3 protein level was associated with increased endothelial cell apoptosis, mirroring evidence of apoptosis susceptibility in PAECs from patients with PAH [21,92]. These in vitro findings were consistent with in vivo findings of increased apoptosis in PAECs in areas of reduced TLR3 staining in diseased human lungs and in rat and mouse models of PH. In our experiments, TLR3 deficiency reduced PAEC migration and this was partially reversed by a caspase inhibitor [21]. Interestingly, the TLR3 agonist, poly(I:C), increased TLR3 expression in rat lung ECs in an IL-10 dependent manner. Prophylactic high-dose poly(I:C) treatment (10 mg/kg three times a week) in the hypoxia and SU5416 PH rat model reduced RVSP and the number of vascular occlusions, but had no significant effect on medial wall thickness or cardiac output [21]. Therapeutic poly(I:C) attenuated established PH when administered 3 weeks after initiation of the disease with hypoxia and SU5416. With both prophylactic and therapeutic poly(I:C) treatment, the number of apoptotic and proliferative cells in the pulmonary arteries were significantly reduced [21].

These data suggest that the TLR3-agonist, poly(I:C), can restore TLR3 levels in TLR3 deficient endothelial cells, thus restoring protective anti-remodeling signals mediated via this pathway. Supporting this hypothesis, other work has revealed protective effects of poly(I:C) that are associated with alterations in apoptosis susceptibility. In the brain, poly(I:C) reduced infarct volume by 57.2% compared to untreated mice subjected to an ischemic/reperfusion injury [93]. This protection was dependent upon TLR3 and was associated with reduced apoptosis in microglial cells [93]. However, there are potentially detrimental consequences associated with activating double-stranded RNA signaling.

## 6. Potential Adverse Effects of TLR3 Activation

### 6.1. Endothelial Dysfunction

Zimmer et al. (2011) found that intravenous poly(I:C) impaired maximal endothelium-dependent vasodilation of aortic segments from wild-type but not TLR3-deficient mice. Poly(I:C) also impaired aortic re-endothelialization after carotid artery injury (electrical denudation) in wild-type mice and aggravated atherosclerotic plaque development in ApoE-deficient mice that were fed high fat diet [94]. These detrimental effects of poly(I:C) are in contrast to the findings by Cole et al. (2011) discussed above, but methodological differences raise some interesting questions about the potential mechanisms. For example, the studies used different methods to injure the vessels, with the carotid cuff model [18] likely to produce less endothelial layer damage than electrical denudation that was used in the study by Zimmer et al. [94]. Intact endothelial and/or medial layers could be important in determining TLR3-mediated responses and models using tissue-specific TLR3-deficient mice may help answer such questions.

Our finding that poly(I:C) suppressed apoptosis in animal models of pulmonary hypertension also contrasts with responses to dsRNA in endothelial cells from other vascular beds in vitro. Poly(I:C) induced TLR3-dependent apoptosis in human umbilical vein cord endothelial cells (HUVECs) in a dose-dependent manner [95]. In addition, poly(I:C) suppressed proliferation and induced apoptosis of human cord blood-derived endothelial progenitor cells (EPC) [96], while another study found similar effects on EPC proliferation but no evidence of apoptosis [97]. Proposed mechanisms linking TLR3 with pro-apoptotic pathways include recruitment of a caspase-8 complex [98,99] and IRF-3 mediated activation of the pro-apoptotic protein, Bax [100]. Independent of changes in cell viability, poly(I:C) has been found to increase endothelial monolayer permeability through upregulation of VEGF signaling [101], changes in calcium flux [102] and altered distribution of tight junction proteins such as claudin-5 [103]. Although these studies link dsRNA-induced TLR3 activation with endothelial dysfunction, these studies were performed using healthy ECs, whereas the endothelial phenotype and function of ECs is fundamentally altered in PAH towards hyperproliferation and apoptosis-resistance. However, as dsRNA induces anti-viral cytokine production, the association between PAH and elevated cytokine levels, notably interferons, requires careful consideration [104].

### 6.2. Interferons and PAH

Double-stranded RNA recognition via TLR3 is a potent inducer of type I IFNs which act in a paracrine manner to elicit anti-viral responses. However, excessive type I IFN activation may contribute to the pathogenesis of autoimmune diseases [105]. The strong association between PAH and autoimmune diseases such as systemic lupus erythematosus and systemic sclerosis also implicates excess IFN activity in PAH pathogenesis. Exogenous α or β-IFN therapy for diseases such as hepatitis C and multiple sclerosis have also been linked to PAH [106,107,108]. Furthermore, patients with known PAH who were subsequently treated with IFN showed changes in hemodynamics and exercise capacity, with the majority having a rise in PVR and fall in 6-min walk distance [106]. In most of the patients, these parameters improved when IFN was discontinued. Studies such as these contributed to type I IFNs being identified as having a ‘possible association’ with PAH at the Fifth World Symposium on PH in 2013 [109]. However, a key limitation with these data is that patients receiving IFN therapy often have co-morbidities that are also risk factors for PAH, such as liver cirrhosis. Additionally, the reversibility of type I-interferon-induced pulmonary hypertension contrasts with the irreversible nature of the disease in other forms of PAH. Nonetheless, mechanistic studies provide plausible connections between IFN and vascular remodeling.

George et al. (2014) subjected IFN-alpha receptor 1 knockout (IFNAR1^-/-^) and wild-type mice to chronic hypoxia and found that IFNAR1^-/-^ mice had lower RVSP, muscularized pulmonary vessels and serum endothelin-1 levels [110]. Human PAH lung tissue had increased IFNAR1 protein expression and circulating levels of the interferon inducible cytokine, IP-10 (or CXCL10) were elevated in patients with systemic sclerosis who had PAH, but not in those without PAH [110]. Interestingly, no differences in circulating IFN-α or -βlevels were found between these patient groups, but there were correlations between hemodynamic indices and IP-10 levels [110]. The same group showed that poly(I:C) induced IP-10 and endothelin-1 release from human PASMCs and this was enhanced when cells were primed with TNF-α [84]. Endothelin-1 levels are elevated in the circulation and lung tissue in PAH and endothelin receptor antagonists are first line oral agents used to treat the disease [111,112].

However, contradictory findings were revealed by Bauer et al. (2014), in a study showing prevention and reversal of PAH by INF-α (human recombinant interferon alpha 2b) in two rodent models (hypoxic mouse model and the SU5146 plus hypoxia rat model). This work also demonstrated suppression of human PAEC and PASMC proliferation by IFN-α in vitro and provided evidence of reduced pulmonary vascular cell proliferation in vivo [113]. These findings are consistent with other work showing attenuated proliferation of human vascular smooth muscle cells when treated with IFN-β [114]. While these studies show evidence of a direct suppressive effect of IFN signaling on human vascular cell proliferation, contrasting data have been reported following stimulation of vascular smooth muscle cells with poly(I:C). Yang et al. (2006) revealed pro-proliferative effects of dsRNA in human coronary artery SMCs that were associated with induction of interleukin-1α [115]. Interestingly, these effects were not observed in mouse aortic smooth muscle cells, implying species-specific differences in dsRNA responses [115] and this is undoubtedly important when considering the potential of dsRNA administration as a therapeutic strategy to reduce pulmonary vascular remodeling.

## 7. Conclusions

This review highlighted the immunoregulatory properties of dsRNA signaling in vascular health and discussed the role of poly(I:C) as an immunomodulator that exerts protective effects in rodent models of PH. Gaps in our knowledge of dsRNA signaling need to be filled before exploring poly(I:C) as a therapy in PAH. It is not known whether the protective effects of synthetic dsRNA in the SU5416 and hypoxia rodent models were mediated via TLR3 signaling in the pulmonary vascular tissue or whether other TLR3-expressing perivascular or circulating leukocytes are important. Furthermore, activation of TLR3 signaling has been associated with upregulation of other dsRNA sensors [116]. Yet how RLRs or PKR signaling contribute to protective vascular phenotypes in PAH has not been investigated. PKR, for example, could have important links to regulatory mechanisms involved in pulmonary vascular remodeling as mutations in one of the other integrated stress response kinases, EIF2AK4, are associated with pulmonary veno-occlusive disease [117].

This review also highlighted the finely tuned balance between protective innate immune responses to dsRNA and potentially detrimental pro-inflammatory signals from receptors activated by the same stimulus. For example, TLR3 deficiency has been linked to altered cell phenotypes in PAH, yet overactivity of this pathway or excessive production of downstream effectors such as IFN, endothelin-1, and pro-inflammatory cytokines may result in EC dysfunction and could thus contribute to PH pathogenesis.

The complexity of the relationship between vascular protection mediated by dsRNA and adverse associations between pulmonary vascular disease and interferon treatment casts doubt on whether targeting this pathway will translate as a treatment for PAH. However, strategies to enhance TLR3 signaling could be developed with synergistic targeting of excessive IFN activity. Now that dsRNA receptor agonists, such as rintatolimod (Ampligen), are emerging in other contexts as safe adjuvants for cancer therapies and for other conditions such as chronic fatigue syndrome [118,119], we believe that exploration of dsRNA as a therapy merits further evaluation in cellular and animal models of PAH.
